# Factors that influence mental health of university and college students in the UK: a systematic review

**DOI:** 10.1186/s12889-022-13943-x

**Published:** 2022-09-20

**Authors:** Fiona Campbell, Lindsay Blank, Anna Cantrell, Susan Baxter, Christopher Blackmore, Jan Dixon, Elizabeth Goyder

**Affiliations:** University of Sheffield, Sheffield, UK

**Keywords:** Student mental health, Mental wellbeing, Risk factors, Rapid review

## Abstract

**Background:**

Worsening mental health of students in higher education is a public policy concern and the impact of measures to reduce transmission of COVID-19 has heightened awareness of this issue. Preventing poor mental health and supporting positive mental wellbeing needs to be based on an evidence informed understanding what factors influence the mental health of students.

**Objectives:**

To identify factors associated with mental health of students in higher education.

**Methods:**

We undertook a systematic review of observational studies that measured factors associated with student mental wellbeing and poor mental health. Extensive searches were undertaken across five databases. We included studies undertaken in the UK and published within the last decade (2010–2020). Due to heterogeneity of factors, and diversity of outcomes used to measure wellbeing and poor mental health the findings were analysed and described narratively.

**Findings:**

We included 31 studies, most of which were cross sectional in design. Those factors most strongly and consistently associated with increased risk of developing poor mental health included students with experiences of trauma in childhood, those that identify as LGBTQ and students with autism. Factors that promote wellbeing include developing strong and supportive social networks. Students who are prepared and able to adjust to the changes that moving into higher education presents also experience better mental health. Some behaviours that are associated with poor mental health include lack of engagement both with learning and leisure activities and poor mental health literacy.

**Conclusion:**

Improved knowledge of factors associated with poor mental health and also those that increase mental wellbeing can provide a foundation for designing strategies and specific interventions that can prevent poor mental health and ensuring targeted support is available for students at increased risk.

**Supplementary Information:**

The online version contains supplementary material available at 10.1186/s12889-022-13943-x.

## Background

Poor mental health of students in further and higher education is an increasing concern for public health and policy [1–4]. A 2020 Insight Network survey of students from 10 universities suggests that “1 in 5 students has a current mental health diagnosis” and that “almost half have experienced a serious psychological issue for which they felt they needed professional help”—an increase from 1 in 3 in the same survey conducted in 2018 [5]. A review of 105 Further Education (FE) colleges in England found that over a three-year period, 85% of colleges reported an increase in mental health difficulties [1]. Depression and anxiety were both prevalent and widespread in students; all colleges reported students experiencing depression and 99% reported students experiencing severe anxiety [5, 6]. A UK cohort study found that levels of psychological distress increase on entering university [7], and recent evidence suggests that the prevalence of mental health problems among university students, including self-harm and suicide, is rising, [3, 4] with increases in demand for services to support student mental health and reports of some universities finding a doubling of the number of students accessing support [8]. These common mental health difficulties clearly present considerable threat to the mental health and wellbeing of students but their impact also has educational, social and economic consequences such as academic underperformance and increased risk of dropping out of university [9, 10].

Policy changes may have had an influence on the student experience, and on the levels of mental health problems seen in the student population; the biggest change has arguably been the move to widen higher education participation and to enable a more diverse demographic to access University education. The trend for widening participation has been continually rising since the late 1960s [11] but gained impetus in the 2000s through the work of the Higher Education Funding Council for England (HEFCE). Macaskill (2013) [12] suggests that the increased access to higher education will have resulted in more students attending university from minority groups and less affluent backgrounds, meaning that more students may be vulnerable to mental health problems, and these students may also experience greater challenges in making the transition to higher education.

Another significant change has been the introduction of tuition fees in 1998, which required students to self fund up to £1,000 per academic year. Since then, tuition fees have increased significantly for many students. With the abolition of maintenance grants, around 96% of government support for students now comes in the form of student loans [13]. It is estimated that in 2017, UK students were graduating with average debts of £50,000, and this figure was even higher for the poorest students [13]. There is a clear association between a student’s mental health and financial well-being [14], with “increased financial concern being consistently associated with worse health” [15].

The extent to which the increase in poor mental health is also being seen amongst non-students of a similar age is not well understood and warrants further study. However, the increase in poor mental health specifically within students in higher education highlights a need to understand what the risk factors are and what might be done within these settings to ensure young people are learning and developing and transitioning into adulthood in environments that promote mental wellbeing.

Commencing higher education represents a key transition point in a young person’s life. It is a stage often accompanied by significant change combined with high expectations of high expectations from students of what university life will be like, and also high expectations from themselves and others around their own academic performance. Relevant factors include moving away from home, learning to live independently, developing new social networks, adjusting to new ways of learning, and now also dealing with the additional greater financial burdens that students now face.

The recent global COVID-19 pandemic has had considerable impact on mental health across society, and there is concern that younger people (ages 18–25) have been particularly affected. Data from Canada [16] indicate that among survey respondents, “almost two-thirds (64%) of those aged 15 to 24 reported a negative impact on their mental health, while just over one-third (35%) of those aged 65 and older reported a negative impact on their mental health since physical distancing began” (ibid, p.4). This suggests that older adults are more prepared for the kind of social isolation which has been brought about through the response to COVID-19, whereas young adults have found this more difficult to cope with. UK data from the National Union of Students reports that for over half of UK students, their mental health is worse than before the pandemic [17]. Before COVID-19, students were already reporting increasing levels of mental health problems [2], but the COVID-19 pandemic has added a layer of “chronic and unpredictable” stress, creating the perfect conditions for a mental health crisis [18]. An example of this is the referrals (both urgent and routine) of young people with eating disorders for treatment in the NHS which almost doubled in number from 2019 to 2020 [19]. The travel restrictions enforced during the pandemic have also impacted on student mental health, particularly for international students who may have been unable to commence studies or go home to see friends and family during holidays [20].

With the increasing awareness and concern in the higher education sector and national bodies regarding student mental health has come increasing focus on how to respond. Various guidelines and best practice have been developed, e.g. ‘Degrees of Disturbance’ [21], ‘Good Practice Guide on Responding to Student Mental Health Issues: Duty of Care Responsibilities for Student Services in Higher Education’ [22] and the recent ‘The University Mental Health Charter’ [2]. Universities UK produced a Good Practice Guide in 2015 called “Student mental wellbeing in higher education” [23]. An increasing number of initiatives have emerged that are either student-led or jointly developed with students, and which reflect the increasing emphasis students and student bodies place on mental health and well-being and the increased demand for mental health support: Examples include: Nightline—www.nightline.ac.uk, Students Against Depression—www.studentsagainstdepression.org, Student Minds—www.studentminds.org.uk/student-minds-and-mental-wealth.html and The Alliance for Student-Led Wellbeing—www.alliancestudentwellbeing.weebly.com/.

Although requests for professional support have increased substantially [24] only a third of students with mental health problems seek support from counselling services in the UK [12]. Many students encounter barriers to seeking help such as stigma or lack of awareness of services [25], and without formal support or intervention, there is a risk of deterioration. FE colleges and universities have identified the need to move beyond traditional forms of support and provide alternative, more accessible interventions aimed at improving mental health and well-being. Higher education institutions have a unique opportunity to identify, prevent, and treat mental health problems because they provide support in multiple aspects of students’ lives including academic studies, recreational activities, pastoral and counselling services, and residential accommodation.

In order to develop services that better meet the needs of students and design environments that are supportive of developing mental wellbeing it is necessary to explore and better understand the factors that lead to poor mental health in students.

### Research objectives

The overall aim of this review was to identify, appraise and synthesise existing research evidence that explores the aetiology of poor mental health and mental wellbeing amongst students in tertiary level education. We aimed to gain a better understanding of the mechanisms that lead to poor mental health amongst tertiary level students and, in so doing, make evidence-based recommendations for policy, practice and future research priorities. Specific objectives in line with the project brief were to:

- To co-produce with stakeholders a conceptual framework for exploring the factors associated with poorer mental health in students in tertiary settings. The factors may be both predictive, identifying students at risk, or causal, explaining why they are at risk. They may also be protective, promoting mental wellbeing.
- To conduct a review drawing on qualitative studies, observational studies and surveys to explore the aetiology of poor mental health in students in university and college settings and identify factors which promote mental wellbeing amongst students.
- To identify evidence-based recommendations for policy, service provision and future research that focus on prevention and early identification of poor mental health

## Methodology

### Identification of relevant evidence

The following inclusion criteria were used to guide the development of the search strategy and the selection of studies.

#### Population

We included students from a variety of further education settings (16 yrs + or 18 yrs + , including mature students, international students, distance learning students, students at specific transition points).

#### Context

Universities and colleges in the UK. We were also interested in the context prior to the beginning of tertiary education, including factors during transition from home and secondary education or existing employment to tertiary education.

#### Outcomes

Any factor shown to be associated with mental health of students in tertiary level education. This included clinical indicators such as diagnosis and treatment and/or referral for depression and anxiety. Self-reported measures of wellbeing, happiness, stress, anxiety and depression were included. We did not include measures of academic achievement or engagement with learning as indicators of mental wellbeing.

#### Study design

We included cross-sectional and longitudinal studies that looked at factors associated with mental health outcomes in Table 5.

### Data extraction and quality appraisal

We extracted and tabulated key data from the included papers. Data extraction was undertaken by one reviewer, with a 10% sample checked for accuracy and consistency The quality of the included studies were evaluated using the Newcastle-Ottawa Scale [26] and the findings of the quality appraisal used in weighting the strength of associations and also identifying gaps for future high quality research.

### Involvement of stakeholders

We recruited students, ex-students and parents of students to a public involvement group which met on-line three times during the process of the review and following the completion of the review. During a workshop meeting we asked for members of the group to draw on their personal experiences to suggest factors which were not mentioned in the literature.

### Methods of synthesis

We undertook a narrative synthesis [27] due to the heterogeneity in the exposures and outcomes that were measured across the studies. Data showing the direction of effects and the strength of the association (correlation coefficients) were recorded and tabulated to aid comparison between studies.

### Search strategy

Searches were conducted in the following electronic databases: Medline, Applied Social Sciences Index and Abstracts (ASSIA), International Bibliography of Social Sciences (IBSS), Science,PsycINFO and Science and Social Sciences Ciatation Indexes. Additional searches of grey literature, and reference lists of included studies were also undertaken.

**Fig. 1:**
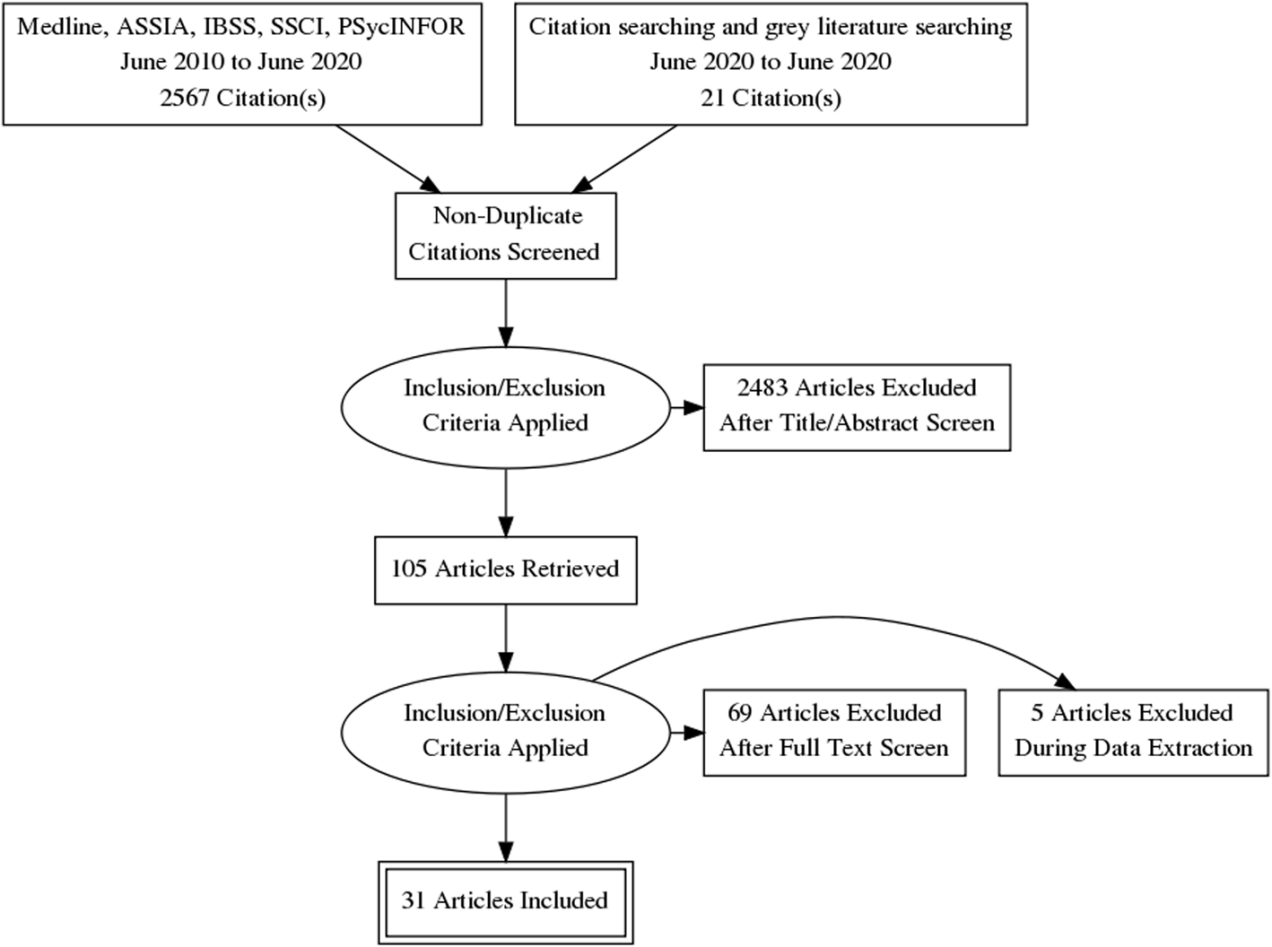
Flow diagram

The search strategy combined a number of terms relating to students and mental health and risk factors. The search terms included both subject (MeSH) and free-text searches. The searches were limited to papers about humans in English, published from 2010 to June 2020. The flow of studies through the review process is summarised in Fig. 1.

The full search strategy for Medline is provided in Appendix 1.

## Results

Thirty-one quantitative, observational studies (39 papers) met the inclusion criteria. The total number of students that participated in the quantitative studies was 17,476, with studies ranging in size from 57 to 3706. Eighteen studies recruited student participants from only one university; five studies (10 publications) [28–37] included seven or more universities. Six studies (7 publications) [35–41] only recruited first year students, while the majority of studies recruited students from a range of year groups. Five studies [39, 42–45] recruited only, or mainly, psychology students which may impact on the generalisability of findings. A number of studies focused on students studying particular subjects including: nursing [46] medicine [47], business [48], sports science [49]. One study [50] recruited LGBTQ (lesbian, gay, bisexual, transgender, intersex, queer/questioning) students, and one [51] recruited students who had attended hospital having self-harmed. In 27 of the studies, there were more female than male participants. The mean age of the participants ranged from 19 to 28 years. Ethnicity was not reported in 19 of the studies. Where ethnicity was reported, the proportion that were ‘white British’ ranged from 71 – 90%. See Table 1 for a summary of the characteristics of the included studies and the participants.

**Table 1:** Table of included studies

Author (year)	Design	Participants	Universities	Year group	Subjects	Female	BAME^a^	IS^a^	Mean age
Berry (2012) [42]	CS -QS	57	1	3.5% (*n* = 2) 1^st^ year 75.4% (*n* = 43) 2nd year 1.8% (*n* = 1) 3^rd^ year 19.3% (*n* = 11) postgraduate	72% psychology	86%	NR	NR	21.2
Boulton (2019) [52]	Longitudinal QS	175	1	49% 1^st^ year 49% 1st year 27% 2nd year 21% 3rd year	multiple	66%	NR	NR	NR
Davies (2019) [53]	longitudinal /prospective	325	NR	NR	NR	70.8%	NR	NR	NR
Denovan (2017a) [43]	longitudinal 1 year	192	1	NR	psychology	82%			19,7
Denovan (2017b) [54]	CS QS	202	1	NR	social science students	73.8%	NR	NR	22.8
El Ansari (2013, 2014 a,b,c 2015) [28–31, 55]	CS QS	3706	7	NR	NR	72.8%	NR	NR	24.9
Freeth(2013) [56]	CS QS	1325	1	NR	NR	61.90%	NR	NR	20.1
Gorczynski (2017)	CS QS	330	1	54.4% 1^st^ year	NR	44.2%	NR	NR	20.9
Gnan(2019) [33]	CS QS	1948	multiple	NR	NR	46.9%	NR	NR	20.3
Hassel (2018) [44]	CS QS	77	1	NR	psychology	80.5%	NR	NR	19.1
Hixenbaugh (2012) [38]	CS QS	429	1	1st year	NR	69% F	NR	NR	21.8
Holliman (2018) [39]	Longitudinal survey	186	1	1st year	psychology	75%	NR	24%	19.2
Honney (2010) [47]	CS QS	853	1	NR	medicine (*n* = 553) non-medical (*n* = 300)	66%	37.4%	NR	?
Jackson (2015) [34]	CS QS	230	multiple	NR	NR	52%	NR	NR	21.3
Jessop (2020) [57]	CS QS	337	1	1^st^ year students, 101 (29.97%) 2nd year, 117 (34.72%) 3rd year and 24 (7.12%) fourth year	NR	69.1%	NR	NR	21.1
Kannanagara (2018) [58]	CS QS (and qualitative interviews)	440	1	81% Undergraduates	NR	55.7%	NR	NR	range 21–39
Kotera (2019) [48]	CS QS	138	1	NR	business	49%	NR	29%	21.2
Lloyd (2014) [59]	CS QS	315	3	NR	NR	83%	8% non-UK born 5% English not primary language	NR	23.4
Mahadevan (2010) [51]	case control	261	1	NR	NR	70%	NR	NR	?
McIntyre (2018) [60]	CS QS	1135	1	• 1^st^ -year students comprised 46% • 2nd—and 3^rd^ year students made up 35% and 21%, respectively	• Health and Life Sciences (30%), Humanities and Social sciences (42%) and Science and Engineering (18%)	71%	18% (non-white Britsih)	NR	20.8
McLafferty (2019) [40]	CS QS	739	4	1^st^ year	NR	61%	NR	NR	21
Nightingale (2013) [41]	longitudinal (1 year)	331	1	1^st^ year	multiple	53.6%	13% non-white British)	NR	18–49
Norbury (2019) [45]	CS QS	546	2	1st 291 (53) 2nd 225 (41) 3rd 30 (6)	89% psychology	84%	N R	NR	20.4
Oliver (2010) [49]	CS QS	146	1	1^st^ or 2nd year	sports science	33.6%	NR	NR	19.3
O'Neill (2018) [50]	longitudinal retrospective (1 year)	739	1	NR	NR	62.5%	NR	NR	21
Por (2011) [46]	prospective correlational survey	130	NR	range	nursing	90%	18.5% African 4% Asian 4% Caribbean	NR	28
Ribchester (2014) [61]	CS QS (and focus group)	413	1	NR	English/Geography	NR	NR	NR	NR
Richardson (2015,2017a,2017b,2018) [35, 36, 62, 63]	longitudinal	390–454	every university	range	NR	77.9%	10%	NR	19.9
Taylor (2020) [64]	CS QS	707	2	NR	multiple faculties	75.2%	17%	NR	23.1
Thomas (2020) [65]	CS QS	510	multiple	1^st^ year	multiple	60.8%	NR	NR	18–24 (*n* = 476)
Tyson (2010) [66]	CS QS	100	1	NR	NR	80%	NR	NR	20.4

*NR* Not reported, CS QS Cross-sectional Questionnaire, Survey ^a^*BAME* (Black, asian, and minority ethnic group), *IS* International students

### Design and quality appraisal of the included studies

The majority of included studies (*n* = 22) were cross-sectional surveys. Nine studies (10 publications) [35, 36, 39, 41, 43, 50–53, 62] were longitudinal in design, recording survey data at different time points to explore changes in the variables being measured. The duration of time that these studies covered ranged from 19 weeks to 12 years. Most of the studies (*n* = 22) only recruited participants from a single university. The use of one university setting and the large number of studies that recruited only psychology students weakens the wider applicability of the included studies.

### Quantitative variables

Included studies (*n* = 31) measured a wide range of variables and explored their association with poor mental health and wellbeing. These included individual level factors: age, gender, sexual orientation, ethnicity and a range of psychological variables. They also included factors that related to mental health variables (family history, personal history and mental health literacy), pre-university factors (childhood trauma and parenting behaviour. University level factors including social isolation, adjustment and engagement with learning. Their association was measured against different measures of positive mental health and poor mental health.

Measurement of association and the strength of that association has some limitations in addressing our research question. It cannot prove causality, and nor can it capture fully the complexity of the inter-relationship and compounding aspect of the variables. For example, the stress of adjustment may be manageable, until it is combined with feeling isolated and out of place. Measurement itself may also be misleading, only capturing what is measureable, and may miss variables that are important but not known. We included both qualitative and PPI input to identify missed but important variables.

The wide range of variables and different outcomes, with few studies measuring the same variable and outcomes, prevented meta-analyses of findings which are therefore described narratively.

The variables described were categorised during the analyses into the following categories:

### Vulnerabilities – factors that are associated with poor mental health

Individual level factors including; age, ethnicity, gender and a range of psychological variables were all measured against different mental health outcomes including depression, anxiety, paranoia, and suicidal behaviour, self-harm, coping and emotional intelligence.

#### Age

Six studies [40, 42, 47, 50, 60, 63] examined a student’s ages and association with mental health. There was inconsistency in the study findings, with studies finding that age (21 or older) was associated with fewer depressive symptoms, lower likelihood of suicide ideation and attempt, self-harm, and positively associated with better coping skills and mental wellbeing. This finding was not however consistent across studies and the association was weak. Theoretical models that seek to explain this mechanism have suggested that older age groups may cope better due to emotion-regulation strategies improving with age [67]. However, those over 30 experienced greater financial stress than those aged 17-19 in another study [63].

#### Sexual orientation

Four studies [33, 40, 64, 68] examined the association between poor mental health and sexual orientation status. In all of the studies LGBTQ students were at significantly greater risk of mental health problems including depression [40], anxiety [40], suicidal behaviour [33, 40, 64], self harm [33, 40, 64], use of mental health services [33] and low levels of wellbeing [68]. The risk of mental health problems in these students compared with heterosexual students, ranged from OR 1.4 to 4.5. This elevated risk may reflect the greater levels of isolation and discrimination commonly experienced by minority groups.

#### Gender

Nine studies [33, 38–40, 42, 47, 50, 60, 63] examined whether gender was associated mental health variables. Two studies [33, 47] found that being female was statistically significantly associated with use of mental health services, having a current mental health problem, suicide risk, self harm [33] and depression [47]. The results were not consistent, with another study [60] finding the association was not significant. Three studies [39, 40, 42] that considered mediating variables such as adaptability and coping found no difference or very weak associations.

#### Ethnicity

Two studies [47, 60] examined the extent to which ethnicity was associated with mental health One study [47] reported that the risks of depression were significantly greater for those who categorised themselves as non-white (OR 8.36 p = 0.004). Non-white ethnicity was also associated with poorer mental health in another cross-sectional study [63]. There was no significant difference in the McIntyre et al. (2018) study [60]. The small number of participants from ethnic minority groups represented across the studies means that this data is very limited.

#### Family factors

Six studies [33, 40, 42, 50, 60] explored the association of a concept that related to a student’s experiences in childhood and before going to university. Three studies [40, 50, 60] explored the impact of ACEs (Adverse Childhood Experiences) assessed using the same scale by Feletti (2009) [69] and another explored the impact of abuse in childhood [46]. Two studies examined the impact of attachment anxiety and avoidance [42], and parental acceptance [46, 59]. The studies measured different mental health outcomes including; positive and negative affect, coping, suicide risk, suicide attempt, current mental health problem, use of mental health services, psychological adjustment, depression and anxiety.

The three studies that explored the impact of ACE’s all found a significant and positive relationship with poor mental health amongst university students. O’Neill et al. (2018) [50] in a longitudinal study (*n* = 739) showed that there was in increased likelihood in self-harm and suicidal behaviours in those with either moderate or high levels of childhood adversities (OR:5.5 to 8.6) [50]. McIntyre et al. (2018) [60] (*n* = 1135) also explored other dimensions of adversity including childhood trauma through multiple regression analysis with other predictive variables. They found that childhood trauma was significantly positively correlated with anxiety, depression and paranoia (ß = 0.18, 0.09, 0.18) though the association was not as strong as the correlation seen for loneliness (ß = 0.40) [60]. McLafferty et al. (2019) [40] explored the compounding impact of childhood adversity and negative parenting practices (over-control, overprotection and overindulgence) on poor mental health (depression OR 1.8, anxiety OR 2.1 suicidal behaviour OR 2.3, self-harm OR 2.0).

Gaan et al.’s (2019) survey of LGBTQ students (*n* = 1567) found in a multivariate analyses that sexual abuse, other abuse from violence from someone close, and being female had the highest odds ratios for poor mental health and were significantly associated with all poor mental health outcomes [33].

While childhood trauma and past abuse poses a risk to mental health for all young people it may place additional stresses for students at university. Entry to university represents life stage where there is potential exposure to new and additional stressors, and the possibility that these students may become more isolated and find it more difficult to develop a sense of belonging. Students may be separated for the first time from protective friendships. However, the mechanisms that link childhood adversities and negative psychopathology, self-harm and suicidal behaviour are not clear [40]. McLafferty et al. (2019) also measured the ability to cope and these are not always impacted by childhood adversities [40]. They suggest that some children learn to cope and build resilience that may be beneficial.

McLafferty et al. (2019) [40] also studied parenting practices. Parental over-control and over-indulgence was also related to significantly poorer coping (OR -0.075 *p* < 0.05) and this was related to developing poorer coping scores (OR -0.21 *p* < 0.001) [40]. These parenting factors only became risk factors when stress levels were high for students at university. It should be noted that these studies used self-report, and responses regarding views of parenting may be subjective and open to interpretation. Lloyd et al.’s (2014) survey found significant positive correlations between perceived parental acceptance and students’ psychological adjustment, with paternal acceptance being the stronger predictor of adjustment.

#### Autism

Autistic students may display social communication and interaction deficits that can have negative emotional impacts. This may be particularly true during young adulthood, a period of increased social demands and expectations. Two studies [56] found that those with autism had a low but statistically significant association with poor social problem-solving skills and depression.

#### Mental health history

Three studies [47, 51, 68] investigated mental health variables and their impact on mental health of students in higher education. These included; a family history of mental illness and a personal history of mental illness.

Students with a family history or a personal history of mental illness appear to have a significantly greater risk of developing problems with mental health at university [47]. Mahadevan et al. (2010) [51] found that university students who self-harm have a significantly greater risk (OR 5.33) of having an eating disorder than a comparison group of young adults who self-harm but are not students.

#### Buffers – factors that are protective of mental wellbeing

##### Psychological factors

Twelve studies [29, 39–43, 46, 49, 54, 58, 64] assessed the association of a range of psychological variables and different aspects of mental wellbeing and poor mental health. We categorised these into the following two categories: firstly, psychological variables measuring an individual’s response to change and stressors including adaptability, resilience, grit and emotional regulation [39–43, 46, 49, 54, 58] and secondly, those that measure self-esteem and body image [29, 64].

The evidence from the eight included quantitative studies suggests that students with psychological strengths including; optimism, self-efficacy [70], resilience, grit [58], use of positive reappraisal [49], helpful coping strategies [42] and emotional intelligence [41, 46] are more likely to experience greater mental wellbeing (see Table 2 for a description of the psychological variables measured). The positive association between these psychological strengths and mental well-being had a positive affect with associations ranging from *r* = 0.2–0.5 and OR1.27 [41, 43, 46, 49, 54] (low to moderate strength of association). The negative associations with depressive symptoms are also statistically significant but with a weaker association (*r* = -0.2—0.3) [43, 49, 54].

**Table 2:** Summary of psychological variables evaluated in the included studies

Variable	Definition
Academic self-efficacy	• a belief in one’s ability to achieve desired results from one’s behaviour in academic settings. Students high in academic self-efficacy perceive tasks, difficulties, and setbacks as challenges to be overcome rather than threats [43, 71]
Adaptability	• the extent to which an individual is able to adjust and modify (manage) cognitive (thoughts), behavioural (actions) and emotional (affective) functioning in the face of changing, novel and uncertain circumstances, situations or conditions [39, 72]
Body image	• the mental image we have of the size, shape and contour of our own bodies as well as of our feelings about these characteristics and the parts that constitute our bodies [29]
Coping	• Strategies adopted to reduce stressors. These can include problem focused approaches or emotion focused strategies and individuals may adopt a variety during the course of a stressful situation [73]
Emotion regulation	• how a person controls, expresses, and manages their emotions which plays a very important role in how they cope and respond to stress [74]
Emotional Intelligence	• type of social intelligence that involves a person's ability to monitor their own and others' emotions, to discriminate among them and to use that information to guide their thinking and actions [75]
Grit	• working strenuously towards challenged, maintaining effort and interest over years despite failure, adversity, and plateaus in progress [76]
Hope	• an individual’s perceived capability to develop a pathway to achieve a goal assumes future outcomes are influenced by goal-oriented cognitions [77]
Optimism	• a generalised positive outcome expectancy, positive expectations that good outcomes will happen, perceive these outcomes as attainable, and persevere in goal-oriented efforts [78]
Positive psychology	• a theoretical approach that focusses on positive individual traits, valued subjective experiences, and positive institutions; it emphasises an understanding of the processes and factors that contribute to the health, success, and flourishing of individuals [79]
Resilience	• the ability to recover from adversity and react adaptively to stressful situations and is a core component of psychological well-being [80]
Self esteem	• the extent to which a person accepts, likes, or is satisfied with themselves [81]
Self-control	• the ability to exercise restraint over behaviour to meet long-term interests [57]
Self-talk	• an intra-personal event that could be interpreted as informational or controlling and may attenuate or exacerbate the negative effects of a stressful experience [82]

Denovan (2017a) [43] in a longitudinal study found that the association between psychological strengths and positive mental wellbeing was not static and that not all the strengths remained statistically significant over time. The only factors that remained significant during the transition period were self-efficacy and optimism, remaining statistically significant as they started university and 6 months later.

#### Parental factors

Only one study [59] explored family factors associated with the development of psychological strengths that would equip young people as they managed the challenges and stressors encountered during the transition to higher education. Lloyd et al. (2014) [59] found that perceived maternal and paternal acceptance made significant and unique contributions to students’ psychological adjustment. Their research methods are limited by their reliance on retrospective measures and self-report measures of variables, and these results could be influenced by recall bias.

#### Self image

Two studies [29, 64] considered the impact of how individuals view themselves on poor mental health. One study considered the impact of self-esteem and the association with non-accidental self-injury (NSSI) and suicide attempt amongst 734 university students. As rates of suicide and NSSI are higher amongst LGBT (lesbian, gay, bisexual, transgender) students, the prevalence of low self-esteem was compared. There was a low but statistically significant association between low self-esteem and NSSI, though not for suicide attempt. A large survey, including participants from seven universities [42] compared depressive symptoms in students with marked body image concerns, reporting that the risk of depressive symptoms was greater (OR 2.93) than for those with lower levels of body image concerns.

#### Mental health literacy and help seeking behaviour

Two studies [48, 68] investigated attitudes to mental illness, mental health literacy and help seeking for mental health problems.

University students who lack sufficient mental health literacy skills to be able to recognise problems or where there are attitudes that foster shame at admitting to having mental health problems can result in students not recognising problems and/or failing to seek professional help [48, 68]. Gorcyznski et al. (2017) [68] found that women and those who had a history of previous mental health problems exhibited significantly higher levels of mental health literacy. Greater mental health literacy was associated with an increased likelihood that individuals would seek help for mental health problems. They found that many students find it hard to identify symptoms of mental health problems and that 42% of students are unaware of where to access available resources. Of those who expressed an intention to seek help for mental health problems, most expressed a preference for online resources, and seeking help from family and friends, rather than medical professionals such as GPs.

Kotera et al. (2019) [48] identified self-compassion as an explanatory variable, reducing social comparison, promoting self-acceptance and recognition that discomfort is an inevitable human experience. The study found a strong, significant correlation between self-compassion and mental health symptoms (*r* = -0.6. *p* < 0.01).

**Fig. 2:**
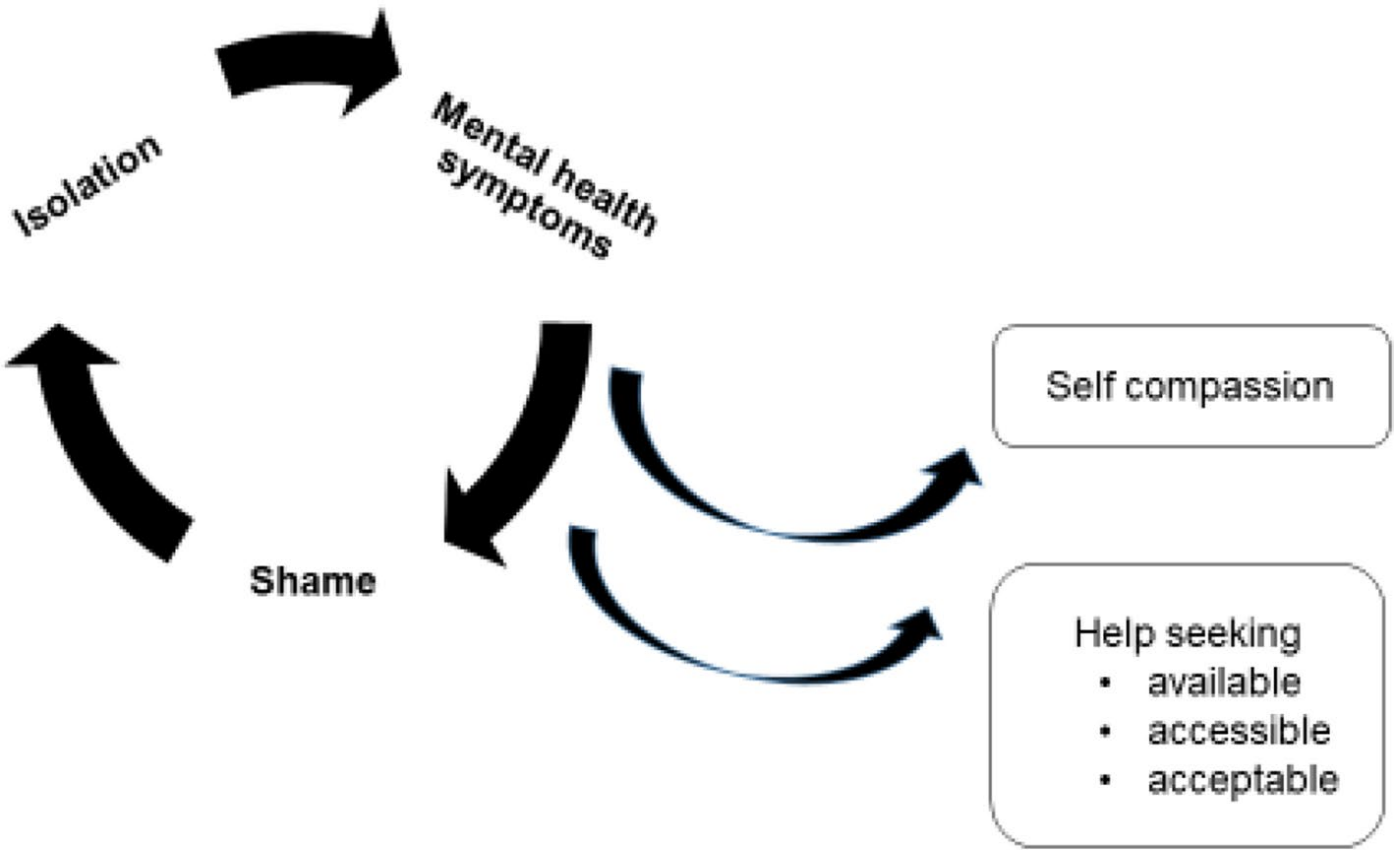
Poor mental health – cycles of reinforcement

There again appears to be a cycle of reinforcement, where poor mental health symptoms are felt to be a source of shame and become hidden, help is not sought, and further isolation ensues, leading to further deterioration in mental health. Factors that can interrupt the cycle are self-compassion, leading to more readiness to seek help (see Fig. 2).

#### Social networks

Nine studies [33, 38, 41, 46, 51, 54, 60, 64, 65] examined the concepts of loneliness and social support and its association with mental health in university students. One study also included students at other Higher Education Institutions [46]. Eight of the studies were surveys, and one was a retrospective case control study to examine the differences between university students and age-matched young people (non-university students) who attended hospital following deliberate self-harm [51].

Included studies demonstrated considerable variation in how they measured the concepts of social isolation, loneliness, social support and a sense of belonging. There were also differences in the types of outcomes measured to assess mental wellbeing and poor mental health. Grouping the studies within a broad category of ‘social factors’ therefore represents a limitation of this review given that different aspects of the phenomena may have been being measured. The tools used to measure these variables also differed. Only one scale (The UCLA loneliness scale) was used across multiple studies [41, 60, 65]. Diverse mental health outcomes were measured across the studies including positive affect, flourishing, self-harm, suicide risk, depression, anxiety and paranoia.

Three studies [41, 60, 62] measuring loneliness, two longitudinally [41, 62], found a consistently positive association between loneliness and poor mental health in university students. Greater loneliness was linked to greater anxiety, stress, depression, poor general mental health, paranoia, alcohol abuse and eating disorder problems. The strength of the correlations ranged from 0–3-0.4 and were all statistically significant (see Tables 3 and 4). Loneliness was the strongest overall predictor of mental distress, of those measured. A strong identification with university friendship groups was most protective against distress relative to other social identities [60]. Whether poor mental health is the cause, or the result of loneliness was explored further in the studies. The results suggest that for general mental health, stress, depression and anxiety, loneliness induces or exacerbates symptoms of poor mental health over time [60, 62]. The feedback cycle is evident, with loneliness leading to poor mental health which leads to withdrawal from social contacts and further exacerbation of loneliness.

**Table 3:** Table of Associations – with Poor Mental Health

	Depression/depressive symptoms	Depression and anxiety / poor MWB	Negative affect/low levels of wellbeing/distress	Anxiety	Paranoia	Self harm	Suicide risk (behaviour)/ideation/attempt	Mental health problems/ use of MH services	Loneliness	Percieved stress	Attachment anxiety /avoidance	Dysfunctional coping / negative engagement
VULNERABILITIES												
< 21	SS** [47] OR 1.8 [40]	NS [60]/ NS [63]		NS [40]	NS [47]	NS [47] OR 0.5	NS/ OR 2.02 [50] > 21 * [40]					-0.27 [42]
LGBTQ (bi vs mono)			SS** [68]			OR 1.4* [33]	OR 1.5** [33]	OR 1.6** [33]				
Non heterosexual	OR 2.2** [40]			OR 2.5** [40]		OR 4.5*** [40]	OR4.2*** [40]					
Trans vs cis						OR 3.0*** [33]	OR 2.4*** [33]	OR 2.8*** [33] / 3.3*** [33]				
LGB (lesbian, gay, bisexual)						OR 1.9* [64]	OR 2.5* [64]					
Ethnicity*	SS** [47]	NS [60] /NS [63]		NS [60]	NS [60]							
Gender (women compared to men)	β 0.09** [60] SS [33, 47]	/NS [63]		β 0.11** [60]	NS [60]	OR 4.0*** [33]	OR 1.3* [33]	OR 2.5** [33] /OR 2.2*** [33]				
Family history of depression	SS*** [47]											
Previous MH problems	SS*** [47]		SS*** [68]									
Eating disorders						OR 5.3* [51]						
Childhood deprivation	β 0.1** [60]			β 0.1** [60]	NS [60]							
Childhood trauma*	β 0.1** [60] SS *—** [40]			β 0.2*** [60] SS ** [40]	β 0.2*** [60]	SS ** [40]SS**[50]	SS ** [40]SS**[50]					
Sexual abuse						OR 1.8** [33]	OR 2.1*** [33]	OR 2.3*** [33] /OR 2.0*** [33]				
Other abuse or violence						OR 2.6*** [33]	OR 2.4*** [33]	OR 1.8*** [33] /OR 2.1*** [33]				
Parental over control x stress	NS [40]			OR 1.1*** [40]		OR 1.1* [40]	NS [40]					
Parental over protection x stress	NS [40]			NS [40]		NS [40]	NS [40]					
Parental over indulgence x stress	OR 1.1*** [40]			OR 1.1*** [40]			OR 1.1* [40]					
Attachment anxiety			NS [42]									
Attachment avoidance			NS [42]									
Perceived parental acceptance											SS** [59]	
Having a disability	*β* 0.14** [63]	β 0.1* [63]		*β* 0.14** [63]								
Social problem solving	*β* 0.5** [35]											
Autism spectrum	*β* 0.4** [34]			r 0.5*** [56]								/ SS*** [56]
BUFFERS												
Response to stress and change												
Self-efficacy/emotional intelligence/ Self compassion/ Adaptability/ Resilience	-SS*** [41]	-SS** [41] r -0.1* [58]	-SS** [41] NS [42] r -0.3*** [43]					r -0.6** [48]	-SS** [41]	r -0.1* [69] r -SS** [48, 54]	NS [43]// r 0.27* [42]	/// r -0.6*** [39]
Optimism, Hope			r -0.3** [43] r -0.2** [43]									
Leisure coping beliefs										r 0.1* [54]		
Engagement in physical activity		r -0.6** [66]										
Self image												
Self-esteem,						SS* [64]	SS* [64] NS [64]					
Body image concerns		OR 2.9 (2.2 to 3.9) [29]									OR 1.3^NR^ [29]	
Developing social networks												
Maintained social capital/ bridging social capital/ Bonding capital									r -0.9 [65], r -0.6** [65] r -0.6** [65]			
Belongingness						r-0.02* [64]						
Controlling self talk			r 0.2* [49]	r 0.3** [49]								
Attitudes to mental health												
Mental health literacy										NS [68]		
Negative attitudes to mental illness			0.11*** [48]									
University factors												
Good induction									-0.6** [65]			
Good experience and understanding of lecture			-SS* [49]	-SS* [49]								
Triggers												
Stress			r 0.5** [43]									
Exams	0.2 [60]											0.27* [42]
Loneliness/ social isolation/ thwarted belongingness	r 0.4*** [60] r 0.4*** [62]			r0.4*** [60] r 0.4*** [62]	r 0.4**** [60]	r 0.9* [60] SS* [64]	SS* [64]			r 0.4*** [62]		-SS** [41]
Relationship difficulties with: 1) parents 2) partners 3) friends						OR 0.5* [51] OR 0.5* [51] OR 2.6* [51]						
Body image concerns	OR 2.9^***^ [29]											
Financial factors	NS [47] NS (T4) [35] SS** [63] *β* 0.3*** [60]	/SS* [83] OR 0.5* [84] NS (T4) [35]		NS (T4) [35] SS** [63] *β* 0.3*** [60]		NS [51]				NS (T4) [35] SS** [63]		
Poor living conditions	SS** [60]			SS** [60]		NS [51]						
RED FLAGS												
Dysfunctional coping			NS [42]									
Unbalanced/unhealthy diet	SS*** [28]		OR 1.7* [84]							SS*** (females only) [28]		
Lack of help seeking			OR 3.7** [84]									
Problem drinking	OR 1.03***/ 1.02*** [31]	/SS^NR^ [38]										
Poor sleep quality				SS*** [45]								
Physical activity		*r-*0.6** [66]		*r-*0.6** [66]								

^***^p < 0.001 **p < 0.01 *p < 0.05

^a^The association between variables were analysed differently in the papers and reported differently. They have included measuring the correlation, hierarchical regression analyses and also calculating the odds of the outcome occurring between groups. This table indicates which studies have measured and reported these associations. Where the reported outcome is measured over several time points, or by gender but remains statistically significant the association is recorded as ‘statistically significant’ (SS). If the value is not reported but is described as statistically significant, then ‘SS’ is also used

Correlations and associations are positive unless indicated with a -

*OR* Odds ratio, *SS* Statistically significant, *NS* Statistically non-significant *T* Time (follow-up point) B: NR: *p* value not reported *r* = correlation coefficient association statistic β = standardized beta which works similarly to a correlation coefficient

*Β* and *r* will range from 0 to 1 or 0 to -1, depending on the direction of the relationship. The closer the value is to 1 or -1, the stronger the relationship

**Table 4:** Table of Associations – with Mental Wellbeing

	Adjustment/ engagement/attachment	Positive affect	Flourishing/ Life satisfaction// wellbeing	Better coping
VULNERABILITIES				
> 21 age*				SS* [40]
Non heterosexual				NS [40]
Gender*—being male	NS [39] NS [42]		// SS* [38]	*β* 0.2*** [40]
Childhood trauma* and moderate stress high risk and stress				NS [40, 60]
Parental acceptance	SS** [59]			NS [40] / SS *^/^** [40]
Attachment anxiety				*β* 0.38** [42]
Attachment avoidance		NS [42]		
BUFFERS				
Psychological strengths Self-efficacy/ emotional management/intelligence, self-control Coping, Grit, Informational self-talk /Resilience/ Adaptability/ self esteem	*β* 0.2** [40] SS** [41] r 0.8** [39]	SS** [43] 0.5 [42] *β* 0.5*** [54] SS** [43] r 0.2* [49]	*β* 0.3*** [54]/ SS** [43]/ 0.3 [46]/ SS** [58]/	SS*** [58]
Optimism Hope		SS** [43] SS** [43]	SS** [43] / SS** [43]	
Mental health literacy			// NS [68]	
Leisure coping beliefs		*β* 0.2* [54]	*β* 0.2** [54]	
Social support				β 0.2*** [40]
Stress		-SS** [43]	/-SS** [43]	
Participation in learning	r 0.8** [39]		/0.2–0.3* [52]	
dysfunctional coping	r -0.4** [42]			
good experience and understanding of lectures		r 0.4** [49]		

Factors associated with protecting against loneliness by fostering supportive friendships and promoting mental wellbeing were also identified. Beliefs about the value of ‘leisure coping’, and attributes of resilience and emotional intelligence had a moderate, positive and significant association with developing mental wellbeing and were explored in three studies [46, 54, 66].

The transition to and first year at university represent critical times when friendships are developed. Thomas et al. (2020) [65] explored the factors that predict loneliness in the first year of university. A sense of community and higher levels of ‘social capital’ were significantly associated with lower levels of loneliness. ‘Social capital’ scales measure the development of emotionally supportive friendships and the ability to adjust to the disruption of old friendships as students transition to university. Students able to form close relationships within their first year at university are less likely to experience loneliness (r-0.09, *r-*0.36, *r-*0.34). One study [38] investigating the relationship between student experience and being the first in the family to attend university found that these students had lower ratings for peer group interactions.

Young adults at university and in higher education are facing multiple adjustments. Their ability to cope with these is influenced by many factors. Supportive friendships and a sense of belonging are factors that strengthen coping. Nightingale et al. (2012) undertook a longitudinal study to explore what factors were associated with university adjustment in a sample of first year students (*n* = 331) [41]. They found that higher skills of emotion management and emotional self-efficacy were predictive of stable adjustment. These students also reported the lowest levels of loneliness and depression. This group had the skills to recognise their emotions and cope with stressors and were confident to access support. Students with poor emotion management and low levels of emotional self-efficacy may benefit from intervention to support the development of adaptive coping strategies and seeking support.

### The positive and negative feedback loops

**Fig. 3:**
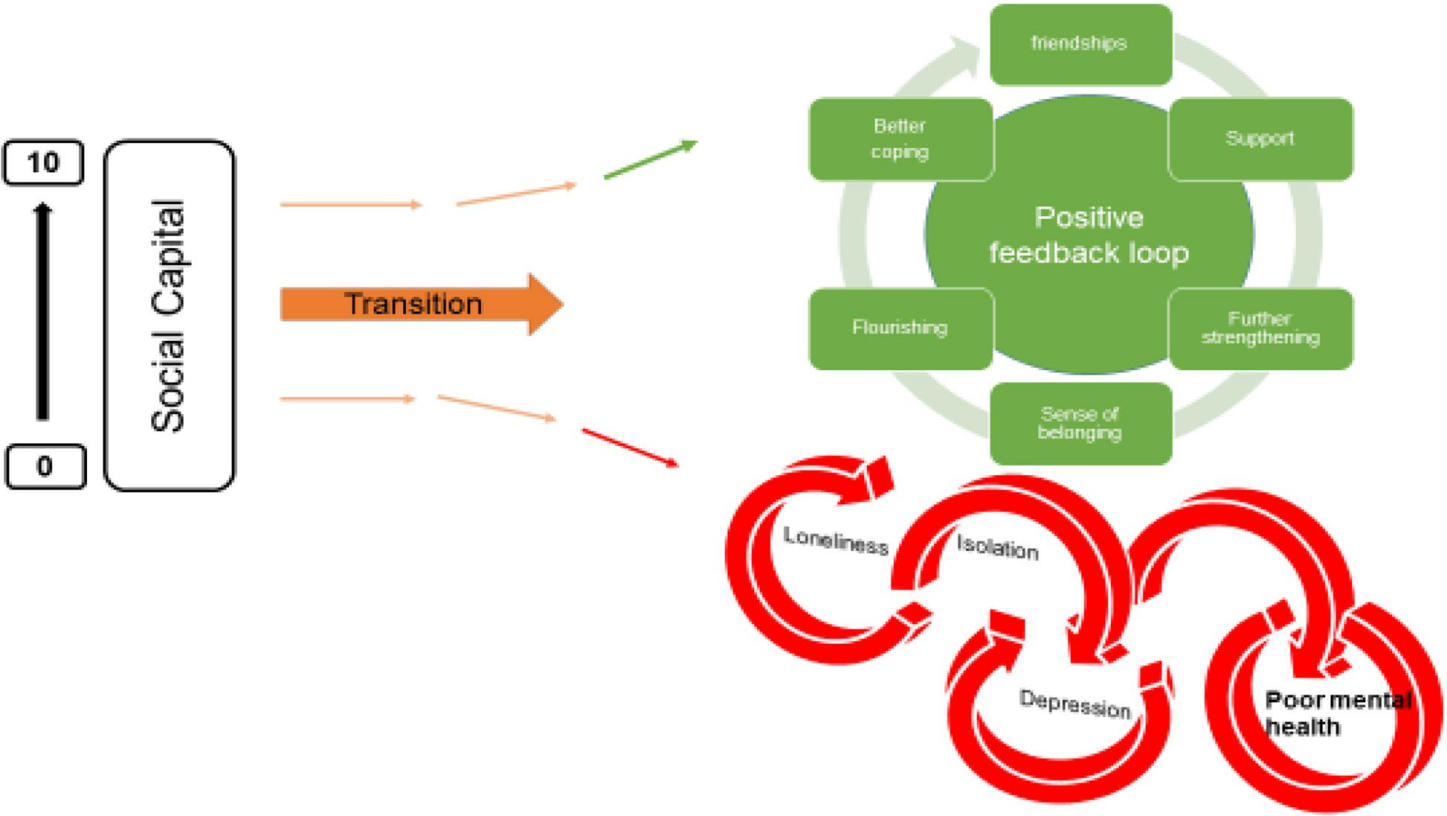
The positive and negative feedback loops

The relationship between the variables described appeared to work in positive and negative feedback loops with high levels of social capital easing the formation of a social network which acts as a critical buffer to stressors (see Fig. 3). Social networks and support give further strengthening and reinforcement, stimulating positive affect, engagement and flourishing. These, in turn, widen and deepen social networks for support and enhance a sense of wellbeing. Conversely young people who enter the transition to university/higher education with less social capital are less likely to identify with and locate a social network; isolation may follow, along with loneliness, anxiety, further withdrawal from contact with social networks and learning, and depression.

### Triggers – factors that may act in combination with other factors to lead to poor mental health

#### Stress

Stress is seen as playing a key role in the development of poor mental health for students in higher education. Theoretical models and empirical studies have suggested that increases in stress are associated with decreases in student mental health [12, 43]. Students at university experience the well-recognised stressors associated with academic study such as exams and course work. However, perhaps less well recognised are the processes of transition, requiring adapting to a new social and academic environment (Fisher 1994 cited by Denovan 2017a) [43]. Por et al. (2011) [46] in a small (*n* = 130 prospective survey found a statistically significant correlation between higher levels of emotional intelligence and lower levels of perceived stress (*r* = 0.40). Higher perceived stress was also associated with negative affect in two studies [43, 46], and strongly negatively associated with positive affect (correlation -0.62) [54].

#### University variables

Eleven studies [35, 39, 47, 51, 52, 54, 60, 63, 65, 83, 84] explored university variables, and their association with mental health outcomes. The range of factors and their impact on mental health variables is limited, and there is little overlap. Knowledge gaps are shown by factors highlighted by our PPI group as potentially important but not identified in the literature (see Table 5). It should be noted that these may reflect the focus of our review, and our exclusion of intervention studies which may evaluate university factors.

**Table 5:** Variables highlighted by the PPI group

• Low morale for BAME groups—‘all the cleaners were black and the lecturers were white’
• Lack of of help with learning basic skills like—how to write essays access research
• Some teaching models make it difficult to integrate
• Loss of support
• Have to take responsibility for your own health
• Peer pressure to say ‘it’s amazing’
• Health and wellbeing services are hard to see, hard to access or unhelpful
• Competitive toxic environments

High levels of perceived stress caused by exam and course work pressure was positively associated with poor mental health and lack of wellbeing [51, 52, 54]. Other potential stressors including financial anxieties and accommodation factors appeared to be less consistently associated with mental health outcomes [35, 38, 47, 51, 60, 62]. Important mediators and buffers to these stressors are coping strategies and supportive networks (see conceptual model Appendix 2). One impact of financial pressures was that students who worked longer hours had less interaction with their peers, limiting the opportunities for these students to benefit from the protective effects of social support.

### Red flags – behaviours associated with poor mental health and/or wellbeing

#### Engagement with learning and leisure activities

**Fig. 4:**
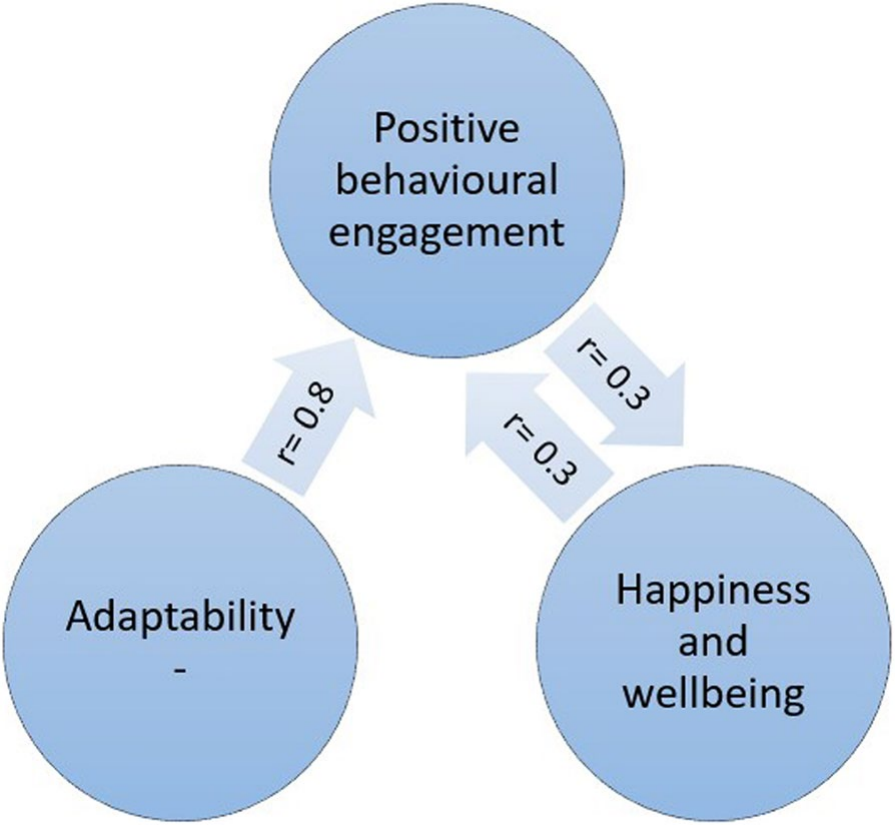
Engagement and wellbeing

Engagement with learning activities was strongly and positively associated with characteristics of adaptability [39] and also happiness and wellbeing [52] (see Fig. 4). Boulton et al. (2019) [52] undertook a longitudinal survey of undergraduate students at a campus-based university. They found that engagement and wellbeing varied during the term but were strongly correlated.

Engagement occurred in a wide range of activities and behaviours. The authors suggest that the strong correlation between all forms of engagement with learning has possible instrumental value for the design of systems to monitor student engagement. Monitoring engagement might be used to identify changes in the behaviour of individuals to assist tutors in providing support and pastoral care. Students also were found to benefit from good induction activities provided by the university. Greater induction satisfaction was positively and strongly associated with a sense of community at university and with lower levels of loneliness [65].

The inte*r-*related nature of these variables is depicted in Fig. 4. Greater adaptability is strongly associated with more positive engagement in learning and university life. More engagement is associated with higher mental wellbeing.

Denovan et al. (2017b) [54] explored leisure coping, its psychosocial functions and its relationship with mental wellbeing. An individual’s beliefs about the benefits of leisure activities to manage stress, facilitate the development of companionship and enhance mood were positively associated with flourishing and were negatively associated with perceived stress. Resilience was also measured. Resilience was strongly and positively associated with leisure coping beliefs and with indicators of mental wellbeing. The authors conclude that resilient individuals are more likely to use constructive means of coping (such as leisure coping) to proactively cultivate positive emotions which counteract the experience of stress and promote wellbeing. Leisure coping is predictive of positive affect which provides a strategy to reduce stress and sustain coping. The belief that friendships acquired through leisure provide social support is an example of leisure coping belief. Strong emotionally attached friendships that develop through participation in shared leisure pursuits are predictive of higher levels of well-being. Friendship bonds formed with fellow students at university are particularly important for maintaining mental health, and opportunities need to be developed and supported to ensure that meaningful social connections are made.

The ‘broaden-and-build theory’ (Fredickson 2004 [85] cited by [54]) may offer an explanation for the association seen between resilience, leisure coping and psychological wellbeing. The theory is based upon the role that positive and negative emotions have in shaping human adaptation. Positive emotions broaden thinking, enabling the individual to consider a range of ways of dealing with and adapting to their environment. Conversely, negative emotions narrow thinking and limit options for adapting. The former facilitates flourishing, facilitating future wellbeing. Resilient individuals are more likely to use constructive means of coping which generate positive emotion (Tugade & Fredrickson 2004 [86], cited by [54]). Positive emotions therefore lead to growth in coping resources, leading to greater well-being.

#### Health behaviours at university

Seven studies [29, 31, 38, 45, 51, 54, 66] examined how lifestyle behaviours might be linked with mental health outcomes. The studies looked at leisure activities [63, 80], diet [29], alcohol use [29, 31, 38, 51] and sleep [45].

Depressive symptoms were independently associated with problem drinking and possible alcohol dependence for both genders but were not associated with frequency of drinking and heavy episodic drinking. Students with higher levels of depressive symptoms reported significantly more problem drinking and possible alcohol dependence [31]. Mahadevan et al. (2010) [51] compared students and non-students seen in hospital for self-harm and found no difference in harmful use of alcohol and illicit drugs.

Poor sleep quality and increased consumption of unhealthy foods were also positively associated with depressive symptoms and perceived stress [29]. The correlation with dietary behaviours and poor mental health outcomes was low, but also confirmed by the negative correlation between less perceived stress and depressive symptoms and consumption of a healthier diet.

Physical activity and participation in leisure pursuits were both strongly correlated with mental wellbeing (*r* = 0.4) [54], and negatively correlated with depressive symptoms and anxiety (*r* = -0.6, -0.7) [66].

## Discussion

Thirty studies measuring the association between a wide range of factors and poor mental health and mental wellbeing in university and college students were identified and included in this review. Our purpose was to identify the factors that contribute to the growing prevalence of poor mental health amongst students in tertiary level education within the UK. We also aimed to identify factors that promote mental wellbeing and protect against deteriorating poor mental health.

Loneliness and social isolation were strongly associated with poor mental health and a sense of belonging and a strong support network were strongly associated with mental wellbeing and happiness. These associations were strongly positive in the eight studies that explored them and are consistent with other meta-analyses exploring the link between social support and mental health [87].

Another factor that appeared to be protective was older age when starting university. A wide range of personal traits and characteristics were also explored. Those associated with resilience, ability to adjust and better coping led to improved mental wellbeing. Better engagement appeared as an important mediator to potentially explain the relationship between these two variables. Engagement led to students being able to then tap into those features that are protective and promoting of mental wellbeing.

Other important risk factors for poor mental wellbeing that emerged were those students with existing or previous mental illness. Students on the autism spectrum and those with poor social problem-solving also were more likely to suffer from poor mental health. Negative self-image was also associated with poor mental health at university. Eating disorders were strongly associated with poor mental wellbeing and were found to be far more of a risk in students at university than in a comparative group of young people not in higher education. Other studies of university students also found that pre-existing poor mental health was a strong predictor of poor mental health in university students [88].

At a family level, the experience of childhood trauma and adverse experiences including, for example, neglect, household dysfunction or abuse, were strongly associated with poor mental health in young people at university. Students with a greater number of ‘adverse childhood experiences’ were at significantly greater risk of poor mental health than those students without experience of childhood trauma. This was also identified in a review of factors associated with depression and suicide related outcomes amongst university undergraduate students [88].

Our findings, in contrast to findings from other studies of university students, did not find that female gender associated with poor mental health and wellbeing, and it also found that being a mature student was protective of mental wellbeing.

Exam and course work pressure was associated with perceived stress and poor mental health. A lack of engagement with learning activities was also associated with poor mental health. A number of variables were not consistently shown to be associated with poor mental health including financial concerns and accommodation factors. Very little evidence related to university organisation or support structures was assessed in the evidence. One study found that a good induction programme had benefits for student mental wellbeing and may be a factor that enables students to become a part of a social network positive reinforcement cycle. Involvement in leisure activities was also found to be associated with improved coping strategies and better mental wellbeing. Students with poorer mental health tended to also eat in a less healthy manner, consume more harmful levels of alcohol, and experience poorer sleep.

This evidence review of the factors that influence mental health and wellbeing indicate areas where universities and higher education settings could develop and evaluate innovations in practice. These include:

- Interventions before university to improve preparation of young people and their families for the transition to university.
- Exploratory work to identify the acceptability and feasibility of identifying students at risk or who many be exhibiting indications of deteriorating mental health
- Interventions that set out to foster a sense of belonging and identify
- Creating environments that are helpful for building social networks
- Improving mental health literacy and access to high quality support services

This review has a number of limitations. Most of the included studies were cross-sectional in design, with a small number being longitudinal (*n* = 7), following students over a period of time to observe changes in the outcomes being measured. Two limitations of these sources of data is that they help to understand associations but do not reveal causality; secondly, we can only report the findings for those variables that were measured, and we therefore have to support causation in assuming these are the only factors that are related to mental health.

Furthermore, our approach has segregated and categorised variables in order to better understand the extent to which they impact mental health. This approach does not sufficiently explore or reveal the extent to which variables may compound one another, for example, feeling the stress of new ways of learning may not be a factor that influences mental health until it is combined with a sense of loneliness, anxiety about financial debt and a lack of parental support. We have used our PPI group and the development of vignettes of their experiences to seek to illustrate the compounding nature of the variables identified.

We limited our inclusion criteria to studies undertaken in the UK and published within the last decade (2009–2020), again meaning we may have limited our inclusion of relevant data. We also undertook single data extraction of data which may increase the risk of error in our data.

## Conclusion

Understanding factors that influence students’ mental health and wellbeing offers the potential to find ways to identify strategies that enhance the students’ abilities to cope with the challenges of higher education. This review revealed a wide range of variables and the mechanisms that may explain how they impact upon mental wellbeing and increase the risk of poor mental health amongst students. It also identified a need for interventions that are implemented before young people make the transition to higher education. We both identified young people who are particularly vulnerable and the factors that arise that exacerbate poor mental health. We highlight that a sense of belonging and supportive networks are important buffers and that there are indicators including lack of engagement that may enable early intervention to provide targeted and appropriate support.

## Supplementary Information

**Additional file 1.**

**Additional file 2.**

## Data Availability

Further details of the study and the findings can be provided on request to the lead author (f.campbell@sheffield.ac.uk).
