# Supplementary material for: Factors that influence mental health of university and college students in the UK: a systematic review
Source: BMC Public Health. 2022 Sep 20;22:1778. doi: 10.1186/s12889-022-13943-x (PMC9484851; doi:10.1186/s12889-022-13943-x)
Supplement: Supplementary file 1 — Additional file 1. [file 12889_2022_13943_MOESM1_ESM.doc]

| **Database** | **Date searched** | **Limits** | **Number of results** |
| --- | --- | --- | --- |
| Medline | 11/06/2020 | English Language, Humans, 2010-Current (June 09, 2020) | 1600 |
| PsycInfo | 11/06/2020 | Human and English Language and yr=”2010-Current” | 4858 |
| ASSIA | 16/06/2020 | English Language, 2010-Current | 1512 |
| IBSS | 16/06/2020 | English Language, 2010-Current | 715 |
| SCI & SSCI | 16/06/2020 | English Language, 2010-2020 | 1438 |
| Total | | | 10,123 |
| **Total after deduplication** | | |  |

Database: Ovid MEDLINE(R) and Epub Ahead of Print, In-Process & Other Non-Indexed Citations, Daily and Versions(R) <1946 to June 09, 2020>

Search Strategy:

--------------------------------------------------------------------------------

1 *Students/ (33349)

2 *Universities/ (15488)

3 1 and 2 (3843)

4 (student$ adj3 (universit$ or "further education" or "higher education" or fe)).ti,ab. (20857)

5 3 or 4 (23461)

6 transition$.ti,ab. (406371)

7 Social Adjustment/ (23224)

8 adjustment$.ti,ab. (192533)

9 Mental Health/ (37794)

10 Mental Disorders/ (161715)

11 (wellbeing or well being).ti,ab. (89221)

12 mental health.ti,ab. (140619)

13 Depression/ (117867)

14 depress$.ab,ti. (454658)

15 Anxiety/ (80200)

16 exp Anxiety Disorders/ (78962)

17 anxiet$.ab,ti. (188858)

18 Suicide/ (39031)

19 suicid$.ab,ti. (76742)

20 6 or 7 or 8 or 9 or 10 or 11 or 12 or 13 or 14 or 15 or 16 or 17 or 18 or 19 (1544126)

21 5 and 20 (4699)

22 correlat$.ab,ti. (1859022)

23 causal factor$.ab,ti. (5495)

24 risk factors/ (818974)

25 factor$.ab,ti. (3332190)

26 Association/ (4009)

27 associat$.ab,ti. (4433600)

28 risk.ab,ti. (2078199)

29 exp Cohort Studies/ (1998151)

30 22 or 23 or 24 or 25 or 26 or 27 or 28 or 29 (9554385)

31 21 and 30 (3225)

32 limit 31 to (english language and humans and yr="2010 -Current") (1600)

33 from 32 keep 1-1600 (1600)

***************************
