# Supplementary figures and images for: Factors that influence mental health of university and college students in the UK: a systematic review
Source: BMC Public Health. 2022 Sep 20;22:1778. doi: 10.1186/s12889-022-13943-x (PMC9484851; doi:10.1186/s12889-022-13943-x)

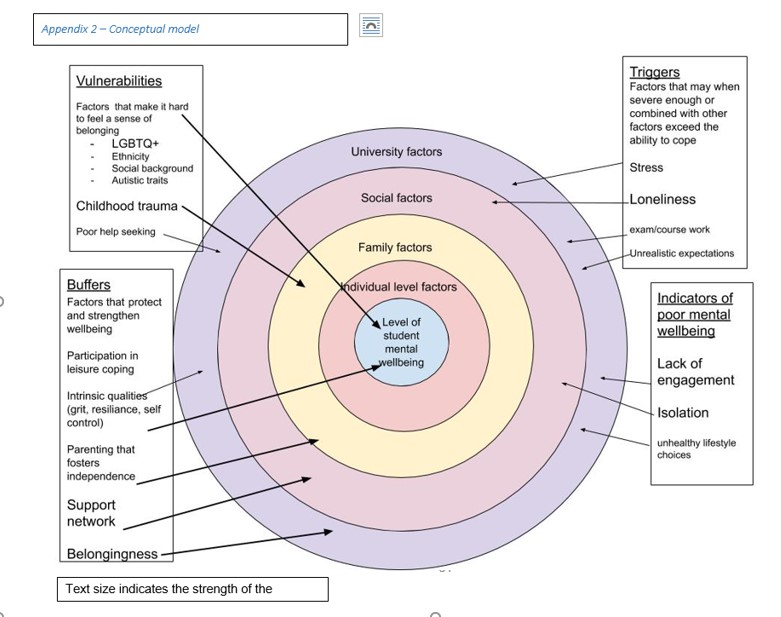

Supplement: Supplementary file 2 — Additional file 2. [file 12889_2022_13943_MOESM2_ESM.jpg]
